# The Yeast Copper Response Is Regulated by DNA Damage

**DOI:** 10.1128/MCB.00116-13

**Published:** 2013-10

**Authors:** Kangzhen Dong, Stephen G. Addinall, David Lydall, Julian C. Rutherford

**Affiliations:** Institute for Cell and Molecular Biosciences, Medical School, Newcastle University, Newcastle upon Tyne, United Kingdom

## Abstract

Copper is an essential but potentially toxic redox-active metal, so the levels and distribution of this metal are carefully regulated to ensure that it binds to the correct proteins. Previous studies of copper-dependent transcription in the yeast Saccharomyces cerevisiae have focused on the response of genes to changes in the exogenous levels of copper. We now report that yeast copper genes are regulated in response to the DNA-damaging agents methyl methanesulfonate (MMS) and hydroxyurea by a mechanism(s) that requires the copper-responsive transcription factors Mac1 and AceI, copper superoxide dismutase (Sod1) activity, and the Rad53 checkpoint kinase. Furthermore, in copper-starved yeast, the response of the Rad53 pathway to MMS is compromised due to a loss of Sod1 activity, consistent with the model that yeast imports copper to ensure Sod1 activity and Rad53 signaling. Crucially, the Mac1 transcription factor undergoes changes in its redox state in response to changing levels of copper or MMS. This study has therefore identified a novel regulatory relationship between cellular redox, copper homeostasis, and the DNA damage response in yeast.

## INTRODUCTION

Copper is an essential enzyme cofactor that is used throughout biology due to its redox properties and its ability to form stable complexes. Established copper enzymes include those involved in respiration, photosynthesis, and the detoxification of reactive oxygen species. Copper is also potentially toxic, since it can displace other metal ions within proteins (1, 2). Copper import and distribution are therefore carefully regulated in all organisms by mechanisms that include the control of gene transcription, the compartmentalization of copper enzymes, the use of chaperones to deliver copper to relevant targets, and the localization and turnover of copper transporters (3–6). Crucial to these processes is the ability of a cell to sense both the availability and the need for copper.

Copper-responsive transcription has been identified in many organisms. Generally, bacteria have no cytoplasmic need for copper, and functionally diverse families of transcription factors regulate its export from the cell (3). In contrast, eukaryotic organisms specifically import copper for its distribution to the mitochondria, to the Golgi apparatus for incorporation into secreted cuproenzymes, and to cytosolic cuproenzymes, including Sod1 and Mek1 (7, 8). In fungi, transcription factors respond to exogenous copper levels and regulate copper import and the sequestration of excess copper in metallothioneins (5). Copper-responsive transcription has also been identified in the alga Chlamydomonas, the plant Arabidopsis, the fly Drosophila melanogaster, and humans (9–13). Our understanding of how transcription factors sense copper differs depending on the organism in question. In bacteria, a major mechanism of metal sensing involves the binding of a metal ion to a relevant transcription factor, and the specificity of response is ensured by a combination of the affinity of the metal ion for its target site and the extent to which metal binding elicits a necessary allosteric change in the sensor (14). Therefore, in bacteria, copper binding to a transcription factor is often the initiating sensing event. In contrast, the molecular basis of copper sensing in eukaryotes is far from understood.

**Fig 1:**
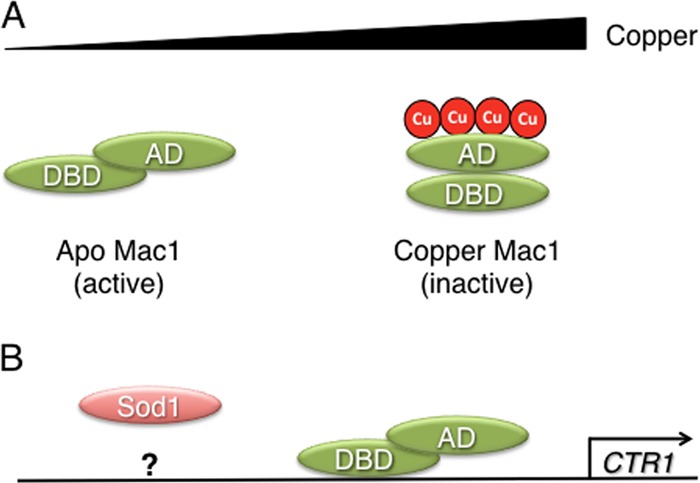
Schematic representation of copper sensing by Mac1. (A) Under low-copper conditions, Mac1 is in an apo form. Copper binds to Mac1 within two cysteine-rich regions in its activation domain (AD), promoting an intramolecular interaction between the AD and the DNA binding domain (DBD). (B) The apo form of Mac1 is the transcriptionally active form, so under low-copper conditions, genes involved in copper uptake are induced. Superoxide dismutase (Sod1) also regulates Mac1 by an unknown mechanism.

In the yeast Saccharomyces cerevisiae, two transcription factors regulate copper gene expression. In response to low-copper conditions, Mac1 activates genes such as *CTR1* and *FRE1*, encoding the copper transporter Ctr1 and the metal reductase Fre1, which together form a high-affinity copper import system (15–18). Conversely, AceI responds to high copper levels and activates the expression of genes that encode copper binding metallothioneins and Sod1 (19–21). The activities of Mac1 and AceI are coordinated so that their target genes are oppositely regulated in response to copper levels (22, 23). Mac1 contains an amino-terminal DNA binding domain and a carboxyl-terminal activation domain and is localized to the nucleus in both copper-replete and copper-starved cells (15, 24–27). Copper regulates Mac1 activity by promoting an intramolecular interaction between the amino- and carboxyl-terminal domains of the protein, thus repressing both its activation domain function and its ability to bind to its target promoters (23–25, 27, 28). The activation domain of Mac1 contains two cysteine-rich regulatory regions (CXCX_4_CXCX_2_CX_2_H), the first of which (the C1 domain) is essential for the repression of Mac1 in response to copper (15, 24, 29). The second domain, C2, has a less-defined role in regulating the DNA binding of Mac1 (28). The finding that both cysteine domains bind 8 molar equivalents of copper *in vitro* supported a model of yeast copper sensing similar to that identified in bacteria (25, 30) (Fig. 1). However, Mac1 has never been isolated from yeast in a copper-bound form, and a recent study established that Mac1 is transcriptionally inactive in mutants that lack Sod1 or its copper chaperone Ccs1 (31). This suggested a potential role for Sod1 in the regulation of Mac1 and raised the question of what constitutes the low-copper-sensing mechanism in yeast.

Using the yeast deletion library, we have identified mutants that exhibit high levels of metal reductase activity on the cell surface. The genes identified include those that function in aspects of genome maintenance, such as those involved in DNA repair and the DNA damage-signaling Rad53 checkpoint pathway. Mutants lacking Ctr1 or Mac1 were sensitive to the DNA-damaging agent methyl methanesulfonate (MMS), suggesting a need for copper during DNA repair. The *CTR1* copper importer and *CUP1* copper metallothionein genes were also regulated in response to MMS by a mechanism that required the Rad53 kinase and the Sod1 copper chaperone Ccs1. Under low-copper conditions, Rad53 signaling was defective due to low Sod1 activity, suggesting the reason why internal copper levels are regulated in response to DNA damage. Mac1 also underwent similar changes in its redox state in response to changes in copper or MMS levels. We have therefore identified a signaling pathway that regulates copper distribution in response to the need for this metal, which alters the paradigm of copper sensing in yeast.

## MATERIALS AND METHODS

### Yeast strains and growth conditions.

All isogenic Saccharomyces cerevisiae deletion strains originated from the BY4741 deletion library, with BY4741 (*MAT***a** *his3*Δ*1 leu2*Δ*0 met15*Δ*0 ura3*Δ*0*) as the wild type (Invitrogen). The *sml1*Δ *rad53*Δ and *sml1*Δ *mec1*Δ mutants were generated by the deletion of *RAD53* and *MEC1*, respectively, in the BY4741 *sml1*Δ strain. The Mac1-Myc and Ctr1-Myc strains were generated by integrating relevant PCR products into the genome of wild-type BY4741 that had been amplified from plasmid pFA6a-13Myc-HISMX6 (32). The SodB plasmid was generated by using homologous recombination to fuse the pRS316 vector with PCR products containing the yeast *SOD1* promoter and terminating sequences and the coding sequence of SodB from Escherichia coli strain K-12 (33). Cells were grown in synthetic dextrose (SD) medium (supplemented with lysine, leucine, methionine, uracil, and histidine) containing 0.25 μM copper (±0.04 μM). The low-copper SD medium (see Fig. 7A) contains 0.14 μM copper (±0.02 μM). Those cultures, treated with methyl methanesulfonate, hydroxyurea (HU), or copper, were grown to an optical density at 595 nm (OD_595_) of 0.5; the compound was added; and cells were grown for an additional hour before being harvested. Low-copper conditions were achieved by continuous growth in the copper chelator bathocuproine disulfonate (BCS) or in a low-copper SD medium (ForMedium). For experiments involving anaerobically grown cells, SD medium was supplemented with ergosterol (10 mg/liter) and Tween 80 (0.05%, vol/vol). For the production of an anaerobic culture, the growth medium was first boiled and then allowed to cool under nitrogen in an anaerobic chamber; cells were added, and the flasks were sealed.

### Screen for metal reductase mutants.

The robotic screen was based on published methods (34). Individual mutants from the deletion library were inoculated into 96-well plates containing liquid yeast extract-peptone-dextrose (YPD) medium from solid agar synthetic genetic array (SGA) plates by using a BioMatrix BM3-09 robot equipped with a 96-pin (1-mm-diameter) pin tool. These mutants were grown to saturation over 2 days, without shaking, at 28°C. The following procedure was then carried out using a Beckman Biomek FX pipetting robot equipped with a magnetic, floating 96-pin pin tool (V&P Scientific, Inc., San Diego, CA). Yeast cultures were resuspended by shaking and were then diluted by dipping pins 3 times into the saturated culture and transferring to a 96-well plate containing sterilized H_2_O in each well; the diluted cultures were then spotted (after further mixing) onto solid YPD agar plates containing the iron chelator bathophenanthroline disulfonate (BPS). The plates were then incubated for 3 days at 30°C, when a red color appeared on those colonies expressing high metal reductase activity on the cell surface. This process was repeated on three separate occasions. Colonies were scored by eye, and care was taken to compare the color of a particular colony with the color of colonies in the same region of the plate. Genes with known functions that were identified in all three replicates were then grouped based on their functions (Yeast GO Slim process) by using the Saccharomyces Genome Database Gene Ontology Slim Mapper (http://www.yeastgenome.org/).

### S1 nuclease protection assays.

Total RNA was isolated from cells by the hot acid phenol method and was hybridized with ^32^P-labeled single-stranded DNA oligonucleotides complementary to the candidate gene and the control gene *CMD1*. The resulting RNA-DNA hybrids were digested with S1 nuclease (Promega), and the samples were electrophoresed through an 8% polyacrylamide–5 M urea gel.

### Protein analysis.

Mid-log-phase cells were treated as indicated in the figure legends, and proteins were isolated using the trichloroacetic acid (TCA) method (35). Reducing SDS-PAGE was performed for normal Western blot analysis, and samples were probed with an anti-Myc antibody (Sigma). In one experiment, samples were treated with alkaline phosphatase (Promega) prior to electrophoresis. To analyze the extent of the modification of thiols in Mac1, the thiol-specific reagent 4-acetamido-4′-maleimidylstilbene-2,2′-disulfonic acid (AMS) (Invitrogen) was used. Following TCA precipitation, protein pellets were resuspended in 100 mM Tris HCl (pH 8.0)–1% SDS–1 mM EDTA containing AMS or, as a control, in 100 mM Tris HCl (pH 8.0)–1% SDS containing 75 mM iodoacetamide (Sigma). Proteins were separated by nonreducing SDS-PAGE and were analyzed by Western blotting. Differences in the level of thiol modification were identified by the extent of reduced protein mobility (0.64 kDa per cysteine residue). In those experiments where the protein was reduced prior to AMS thiol modification, the protein pellet was incubated in the AMS buffer lacking AMS but containing dithiothreitol (DTT) for 1 h. Proteins were then precipitated with TCA, and the AMS protocol was followed.

### Miscellaneous procedures.

For phenotypic analysis, the wild type and the *mac1*Δ mutant were spotted onto YPD plates containing MMS (0.02%). To test superoxide dismutase activity, protein extracts were obtained using the glass bead method and were run on a nondenaturing gel, followed by nitroblue tetrazolium staining (36). Metal reductase activity on the surfaces of liquid-grown cells was quantified as described previously (37).

## RESULTS

### Screen for yeast mutants with high reductase activity on the cell surface.

The yeast Saccharomyces cerevisiae expresses two metal reductases on the cell surface (Fre1 and Fre2) that facilitate the import of iron and copper (38, 39). Mac1 induces *FRE1* expression in response to low copper levels, and the iron-responsive factor Aft1 activates the expression of both *FRE1* and *FRE2* under iron-limiting conditions (39, 40). To identify novel aspects of iron and/or copper metabolism in yeast, we undertook a genetic screen to isolate mutants that exhibit high levels of reductase activity on the cell surface. The levels of metal reductase activity can be quantified using the iron chelator bathophenanthroline disulfonate (BPS), which binds to ferrous (rather than ferric) iron to produce a red chromophore (37). A library of S. cerevisiae deletion mutants was grown on rich medium agar plates containing BPS. Colonies exhibiting high Fre reductase activity were red, whereas wild-type cells and mutants exhibiting normal Fre reductase activity remained a normal color. The genes deleted in those mutants that exhibited higher-than-wild-type levels of Fre reductase activity in three independent experiments were grouped based on their functions.

**Fig 2:**
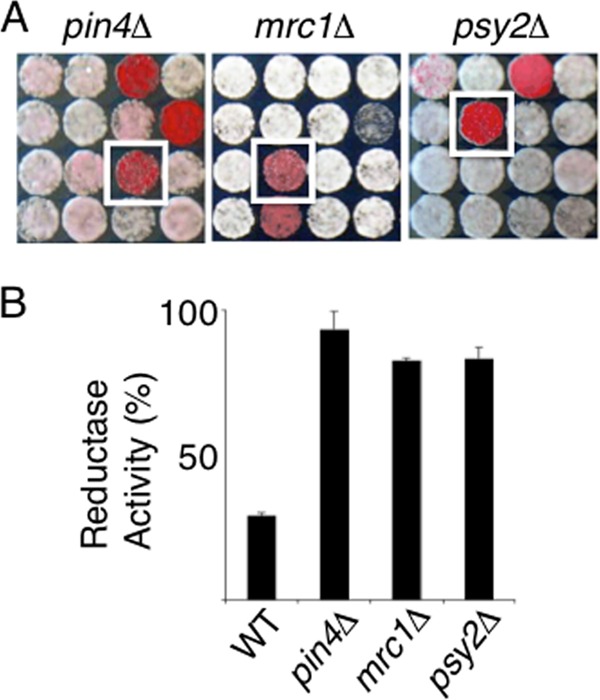
Identification of mutants with high metal reductase activity on the cell surface. (A) The YPD-BPS agar plates on which the yeast deletion collection was grown were inspected visually in order to identify mutants of interest. Those colonies expressing high reductase activity turned red after growth for 3 days at 30°C. Shown are sections of the plates containing the *pin4*Δ, *mrc1*Δ, and *psy2*Δ mutants (boxed in white). (B) Metal reductase activity on the cell surface was quantified in mid-log-phase wild-type (WT), *pin4*Δ, *mrc1*Δ, and *psy2*Δ cells grown in rich YPD medium.

Approximately 250 genes with a variety of known functions were identified in the genetic screen, including those involved in DNA damage, DNA repair, and DNA replication (see Table S1 in the supplemental material). Within this group were three genes (*PIN4*, *MRC1*, and *PSY2*) the products of which influence the regulation of the Rad53 kinase, a downstream effector of the Mec1 checkpoint signal transduction pathway (Fig. 2A). The Mec1 pathway senses a range of genomic stresses, such as double-stranded breaks, replicative stress, and UV damage to nucleotides, and initiates repair mechanisms that include the arrest of the cell cycle, the stabilization of replication forks, and the induction of genes involved in DNA repair (41). Pin4 controls cell cycle progression through its physical interaction with Rad53 (42). Mrc1 is a checkpoint mediator protein that is involved in Rad53 activation in response to replicative stress (43). Psy2 is part of a protein phosphatase complex that regulates Rad53 function to initiate replication fork restart following DNA repair (44). To confirm that these regulators of Rad53 influence metal homeostasis, the levels of metal reductase activity on the surfaces of wild-type, *pin4*Δ, *mrc1*Δ, and *psy*2Δ cells grown in liquid culture were quantified (Fig. 2B). In agreement with the results of our genetic screen, cells lacking Pin4, Mrc1, or Psy2 exhibited higher-than-wild-type levels of metal reductase activity on the cell surface.

The identification of genes involved in genome maintenance and DNA damage sensing suggested a novel connection between these processes and the mechanisms that regulate copper and/or iron homeostasis in yeast. Previous studies have identified links between copper metabolism and genomic stress in yeast. A major target for copper in eukaryotic cells is copper zinc superoxide dismutase (Sod1) (7). Mutants lacking Sod1 activity exhibit defective Rad53 signaling and are sensitive to the DNA-damaging agent hydroxyurea (45). A global analysis of genetic relationships involving synthetic lethality or fitness identified interactions between genes involved in genome maintenance and those encoding Mac1, Sod1, or the Sod1 copper chaperone Ccs1 (46). Mutants that lack Sod1 activity also exhibit enhanced rates of spontaneous mutation under aerobic conditions (47). Based on those previous studies and on the results of our genetic screen, we decided to investigate the link between copper metabolism and the response of yeast cells to DNA damage.

### Copper is required for the resistance of yeast to the DNA-methylating agent methyl methanesulfonate.

**Fig 3:**
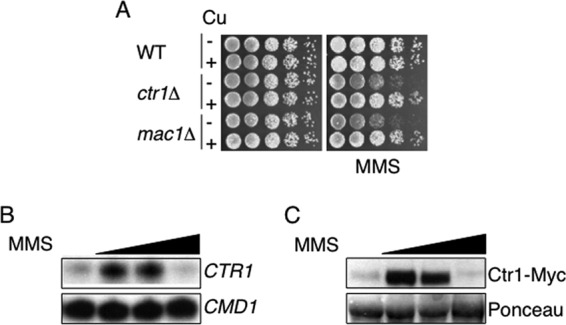
Mac1 is required for the resistance of yeast to MMS. (A) Wild-type, *ctr1*Δ, and *mac1*Δ cells were grown overnight in rich YPD medium with (+) or without (−) copper (100 μM), washed, serially diluted, spotted onto YPD agar plates containing MMS (0.03%), and grown at 30°C. (B) *CTR1* transcripts are regulated in response to MMS. S1 nuclease protection assays were performed using RNA isolated from cells treated with a range of MMS concentrations (0.04, 0.1, 0.3%) for 1 h. Calmodulin was used as a loading control (*CMD1*). (C) Ctr1 protein levels are regulated in response to MMS. Membrane fractions from cells expressing a Ctr1-Myc fusion were treated with a range of MMS concentrations (0.04, 0.1, 0.3%) for 1 h and were used to identify the Myc epitope by Western blotting. Ponceau S staining of the polyvinylidene difluoride membrane was used as a loading control.

The alkylating agent MMS modifies both guanine and adenine nucleotides, to 7-methylguanine and 3-methlyladenine, respectively, and results in the phosphorylation of Rad53 (48, 49). To determine if the sensitivity of yeast to MMS is influenced by copper metabolism, the growth of mutants lacking the high-affinity copper importer Ctr1 or the low-copper-sensing transcription factor Mac1 was analyzed. Wild-type, *ctr1*Δ, and *mac1*Δ cells were grown overnight in a rich medium in the presence or absence of copper, washed, and serially diluted onto agar plates containing MMS (Fig. 3A). The *ctr1*Δ and *mac1*Δ strains were more sensitive to growth on MMS than the wild-type strain (Fig. 3A). For both the *ctr1*Δ and *mac1*Δ strains, copper supplementation improved growth on MMS, in agreement with the higher sensitivity of yeast cells to MMS under copper-limiting conditions (Fig. 3A).

The need for copper for growth on MMS suggested that yeast cells might regulate copper uptake in response to this DNA-damaging agent. To test this hypothesis, the abundance of *CTR1* transcripts was analyzed in cells grown in increasing concentrations of MMS (Fig. 3B). Treatment of cells with 0.04 or 0.1% MMS resulted in increases in *CTR1* transcript levels over those in untreated cells (Fig. 3B). In cells treated with a higher concentration of MMS (0.3%), however, no *CTR1* transcripts could be detected (Fig. 3B). Consistent with the observed changes in the levels of *CTR1* transcripts, the abundance of the Ctr1 protein increased in response to low levels of MMS, but this protein could not be detected in cells treated with the higher concentration of MMS (Fig. 3C). Together, these data are consistent with differential regulation of copper import into yeast in response to increasing levels of DNA methylation.

### The copper chaperone Ccs1 is required for the regulation of yeast copper genes in response to DNA-damaging agents.

**Fig 4:**
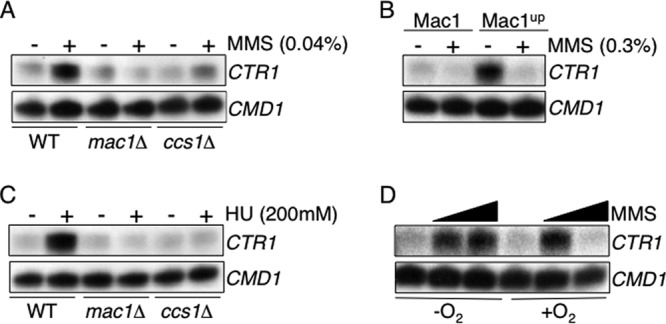
The regulation of *CTR1* in response to MMS requires Mac1, Ccs1, and oxygen. *CTR1* transcripts were analyzed in cells treated with MMS (A and B), hydroxyurea (C), or MMS (0.04 and 0.3%) in the presence or absence of oxygen (D) for 1 h. S1 nuclease protection assays were performed using RNA isolated from wild-type (A, C, and D), *mac1*Δ (A, B, and C), and *ccs1*Δ (A and C) cells and from cells expressing the Mac1^up^ allele (B). Calmodulin was used as a loading control (*CMD1*).

Transcription of the yeast *CTR1* gene is induced in response to low-copper conditions by a mechanism that requires the transcription factor Mac1 and the copper chaperone Ccs1 (31). To determine whether the same proteins are required for the response of *CTR1* to MMS, *CTR1* transcript levels were analyzed in wild-type, *mac1*Δ, and *ccs1*Δ cells that had been treated with MMS (Fig. 4A). The *ccs1*Δ strain lacks Sod1 activity and was used instead of the *sod1*Δ strain because it has a less-severe growth defect, possibly due to the zinc binding capacity of the Sod1 protein and its influence on zinc homeostasis (50). *CTR1* transcript levels increased in wild-type cells treated with MMS but remained low in cells lacking Mac1 or Ccs1 (Fig. 4A). Therefore, in addition to their established role in copper signaling, both Mac1 and Ccs1 are required for the regulation of *CTR1* in response to MMS. A Mac1 genetic variant, Mac1^up^, contains a histidine-to-glutamine substitution generating a factor that is not repressed by copper and that constitutively activates its target genes (15). To test if the Mac1^up^ allele responds to the normally repressing concentration of MMS (0.03%), *CTR1* transcripts were analyzed in cells containing the Mac1^up^ allele and treated with MMS (Fig. 4B). In contrast to the known response of the Mac1^up^ allele to copper, *CTR1* transcripts were repressed in cells expressing the Mac1^up^ allele and treated with high levels of MMS (Fig. 4B).

In addition to being phosphorylated in response to MMS, Rad53 is also modified and activated in response to hydroxyurea, which represses ribonucleotide reductase, preventing the increase in deoxyribonucleotide synthesis that occurs during S phase (49, 51, 52). To determine if *CTR1* responds to a compound that elicits a Rad53 response through a mechanism different from that of MMS, *CTR1* transcript levels were analyzed in cells that had been treated with hydroxyurea (Fig. 4C). Hydroxyurea treatment resulted in the induction of yeast *CTR1* transcripts in wild-type cells but not in cells lacking Mac1 or Ccs1 (Fig. 4C). Therefore, a Mac1- and Ccs1-dependent mechanism regulates yeast *CTR1* transcripts in cells grown under three different conditions (added MMS, added hydroxyurea, or low copper levels).

The need for Ccs1 in the regulation of *CTR1* transcripts in response to MMS and HU suggested that oxygen might be important for the signaling pathway that links DNA damage and copper homeostasis. To test this, *CTR1* transcripts were analyzed in cells that had been treated with MMS and grown under anaerobic or aerobic conditions. In anaerobically grown cells, both low and high levels of MMS resulted in increases in *CTR1* transcript levels (Fig. 4D). In aerobically grown cells, *CTR1* was regulated as described above, with a high level of MMS repressing transcript levels (Fig. 4D). Therefore, oxygen influences the regulation of *CTR1* in response to MMS, since oxygen is required for the repression *CTR1* transcript levels in response to a high level of MMS.

### Rad53 is required for the response of yeast copper genes.

**Fig 5:**
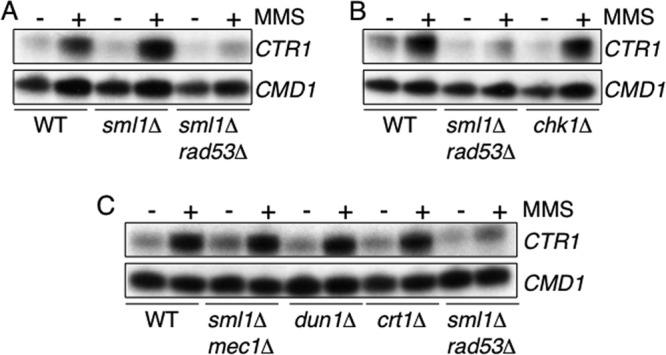
The regulation of *CTR1* in response to MMS requires Rad53 but is independent of other Mec1 pathway components. *CTR1* transcripts were analyzed in cells treated with 0.04% MMS for 1 h by S1 nuclease protection assays using RNA isolated from wild-type (A to C), *sml1*Δ (A), *sml1*Δ *rad53*Δ (A to C), *chk1*Δ (B), *sml1*Δ *mec1*Δ, *dun1*Δ, and *crt1*Δ (C) cells. Calmodulin was used as a loading control (*CMD1*).

The response of *CTR1* to MMS and the need of yeast for copper during growth on MMS suggested that the yeast copper genes might be regulated as part of the response of yeast cells to DNA damage. Our finding that proteins that regulate Rad53 function are required for the regulation of reductase activity on the cell surface prompted us to analyze the potential role of this kinase in the regulation of *CTR1*. Rad53 is an essential protein; however, the *RAD53* gene can be deleted in combination with *SML1*, which codes for an inhibitor of ribonucleotide reductase (53). Analysis of the response of the *RNR3* gene to MMS, a downstream target of Rad53, and Western blot analysis of relevant cell extracts confirmed the lack of Rad53 activity in our *sml1*Δ *rad53*Δ strain (data not shown). To determine if Rad53 is required for the response of *CTR1* to MMS, transcript levels were analyzed in wild-type, *sml1*Δ, and *sml1*Δ *rad53*Δ cells treated with MMS. In *sml1*Δ cells, *CTR1* transcripts were regulated as described above in wild-type cells and were induced in response to MMS (Fig. 5A). In cells lacking Rad53, however, *CTR1* was not induced in response to MMS (Fig. 5A). The profile of *CTR1* expression in response to MMS in cells lacking Rad53 was therefore similar to that identified in the *ccs1*Δ mutant (Fig. 4A).

The Mec1 checkpoint protein regulates the distinct Rad53 and Chk1 pathways (54, 55). To test if Chk1 is also involved in the regulation of copper import, the response of *CTR1* in cells lacking Chk1 was analyzed. The analysis established that Chk1 is not required for the regulation of *CTR1* in response to MMS (Fig. 5B). To further analyze the role of the Mec1 pathway in the regulation of copper import, *CTR1* transcript levels were also analyzed in mutants lacking the Mec1, Dun1, and Crt1 components of the Mec1 pathway, which are involved in the regulation of the ribonucleotide reductase genes (*RNR2*, *RNR3*, and *RNR4* [*RNR* genes]) in response to DNA damage (56). In cells lacking Mec1, Dun1, or Crt1 and treated with MMS, *CTR1* exhibited a wild-type transcriptional profile, establishing that these proteins are not required for the regulation of *CTR1* in response to MMS (Fig. 5C). Therefore, the signal transduction pathway that regulates *CTR1* in response to MMS is distinct from that which regulates the *RNR* genes.

### Copper is required for the functioning of the Rad53 pathway.

**Fig 6:**
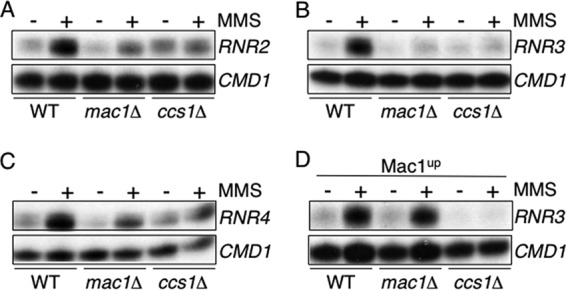
The response of the ribonucleotide genes (*RNR2*, *RNR3*, and *RNR4*) to MMS requires Ccs1 and Mac1. *RNR* transcripts were analyzed in cells grown with the copper chelator BCS (100 μM) and were then treated with 0.04% MMS for 1 h by S1 nuclease protection assays using RNA isolated from wild-type, *mac1*Δ, and *ccs1*Δ cells (A to D) and from cells expressing the Mac1^up^ allele (D). Calmodulin was used as a loading control (*CMD1*).

The induction of copper import in response to DNA damage was unexpected, since it might be predicted that the levels of a metal that can catalyze the production of the hydroxyl radical would be reduced when genomic stress was sensed. A previous study has shown that the Rad53 pathway is compromised in cells lacking Sod1 activity (45). We hypothesized that the Rad53 pathway would not function under low-copper conditions due to a reduction in Sod1 activity. To test this model, transcripts of the Rad53-regulated *RNR* genes were analyzed in wild-type, *mac1*Δ, and *ccs1*Δ cells that had been grown in the presence of the copper chelator bathocuproine disulfonate (BCS) and treated with MMS. In wild-type cells, the *RNR* transcripts were fully induced in response to MMS; however, in cells lacking Mac1, the *RNR* transcripts were only partially induced (Fig. 6A, B, and C). In cells lacking Ccs1, the *RNR* transcripts exhibited a low constitutive level of expression (Fig. 6A, B, and C). Therefore, the growth of the *mac1*Δ and *ccs1*Δ mutants under copper-limiting conditions results in a defect in the regulation of the Rad53 downstream targets *RNR2*, *RNR3*, and *RNR4*. To test if the constitutive expression of copper import could reverse this phenotype, *RNR3* transcript levels were analyzed in cells expressing the Mac1^up^ allele (Fig. 6D). As expected, the Mac1^up^ allele restored wild-type levels of the *RNR3* transcripts in the *mac1*Δ strain. However, the constitutively active Mac1^up^ allele failed to restore signaling in the *ccs1*Δ mutant, in agreement with the notion that copper delivery to Sod1 is required for the regulation of *RNR3* (Fig. 6D).

**Fig 7:**
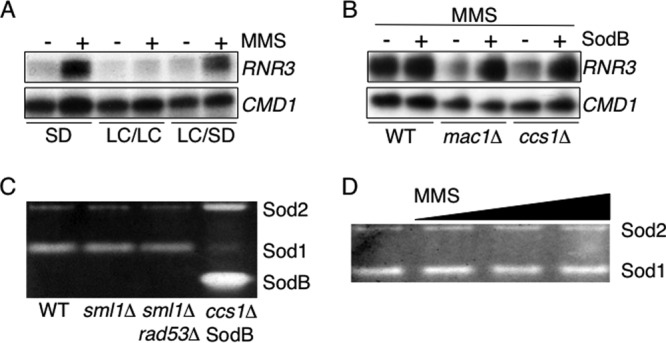
The Rad53 pathway requires copper in order to function. (A) *RNR3* transcripts were analyzed by S1 nuclease protection assays using RNA isolated from *mac1*Δ cells grown in synthetic dextrose medium (SD) or from *mac1*Δ cells grown in a low-copper synthetic medium (LC) for 5 h and then divided between LC medium (LC/LC) and SD medium (LC/SD) prior to treatment with 0.04% MMS for 1 h. Calmodulin was used as a loading control (*CMD1*). (B) The experiment was conducted as for panel A except that all cells were grown in LC medium for 5 h prior to treatment with 0.04% MMS for 1 h and contained either an empty vector or a plasmid expressing bacterial SodB. Sod1 activity does not change in a mutant lacking Rad53 or in response to MMS. (C and D) Extracts from wild-type, *sml1*Δ, and s*ml1*Δ *rad53*Δ cells (C) and from wild-type cells treated with a range of MMS concentrations (0.04, 0.1, 0.3%) (D) were assayed for superoxide dismutase activity using nitroblue tetrazolium staining. The *ccs1*Δ strain containing bacterial SodB was also analyzed to confirm SodB activity (C).

To confirm that the lack of wild-type *RNR3* expression in the *mac1*Δ and *ccs1*Δ strains was due to low-copper conditions, *RNR3* transcripts were quantified in the *mac1*Δ strain grown in media containing different levels of copper. Synthetic dextrose (SD) medium contains sufficient copper to repress *CTR1*, so yeast cells grown under these conditions are not copper starved, whereas growth of yeast in a low-copper SD medium results in the induction of *CTR1* expression (23). *RNR3* transcripts were induced in response to MMS in the *mac1*Δ strain grown in a copper-replete SD medium, as would be expected in wild-type cells (Fig. 7A, 2nd lane). To test the influence of low copper levels on the regulation of *RNR3*, the *mac1*Δ strain was grown in a low-copper medium to deplete copper stores, harvested, divided, and then inoculated into fresh low-copper and copper-replete SD media and grown for an additional hour. *RNR3* transcripts were not induced in response to MMS in cells starved of copper but were identified in those cells that had been returned to a copper-replete medium (Fig. 7A, 3rd to 6th lanes). Copper is therefore needed for the wild-type regulation of the Rad53 target *RNR3* in cells exposed to MMS.

We speculated that the loss of Rad53 signaling under low-copper conditions might be reversed by the activity of a superoxide dismutase enzyme that contains a metal other than copper at its active site. Restoration of Rad53 under these conditions would establish a link between copper, superoxide dismutase activity, and Rad53 function. Escherichia coli SodB is an iron-containing superoxide dismutase that has not, to our knowledge. been used previously in S. cerevisiae studies (57). To determine if the loss of *RNR3* regulation under low-copper conditions is due to the loss of superoxide dismutase activity, we analyzed the extent to which SodB can restore wild-type *RNR3* expression in copper-starved yeast. In cells lacking Mac1 or Ccs1 that were grown under low-copper conditions, MMS treatment failed to induce *RNR3* transcripts to the level of those detected in wild-type cells, as described above (Fig. 7B). However, *RNR3* regulation was restored in the *mac1*Δ and *ccs1*Δ mutant strains when SodB was expressed from a low-copy-number plasmid (Fig. 7B). Analysis of superoxide dismutase activity in relevant cell extracts confirmed that SodB is active in our experimental system (Fig. 7C). Taken together, these data are consistent with the notion that the Rad53 pathway is inactive under copper-limiting conditions due to a loss of Sod1 activity.

The need for Sod1 activity for the response of *CTR1* to MMS suggested that *CTR1* might be regulated in response to changes in Sod1 activity. To test this model, Sod1 activity was analyzed in wild-type, *sml1*Δ, and *sml1*Δ *rad53*Δ cells and was found to be equivalent in these strains (Fig. 7C). Sod1 activity levels also remained constant in cells treated with MMS both at concentrations that activate *CTR1* transcription and at concentrations that repress *CTR1* transcription (Fig. 7D). Therefore, there is no correlation between changes in the abundance of *CTR1* transcripts and Sod1 activity.

### The redox state of Mac1 changes in response to MMS.

In addition to activating the Rad53 pathway, MMS and hydroxyurea also induce the yeast transcription factor Yap1, which regulates genes in response to oxidative stress (58, 59). The activity of Yap1 is regulated by the reversible oxidation of regulatory cysteine residues, which alters the interaction of Yap1 with the nuclear export receptor Crm1, thereby regulating Yap1 nuclear localization (60, 61). The fact that Yap1 and Mac1 contain regulatory cysteine residues and respond to MMS and hydroxyurea suggested that similar molecular mechanisms might regulate these factors.

**Fig 8:**
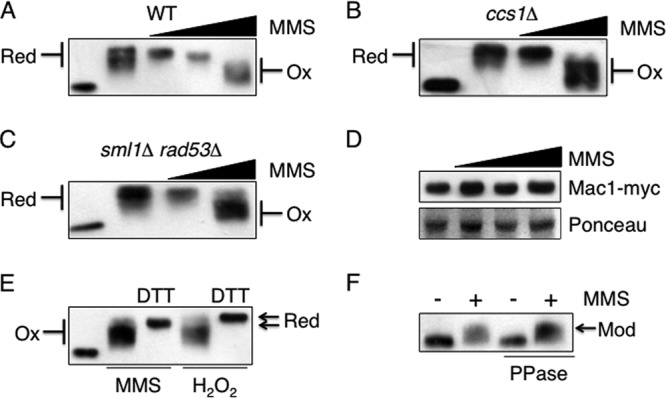
Mac1 is modified and undergoes changes in its redox state in response to MMS. (A to C) The oxidation state of Mac1 was analyzed by nonreducing SDS-PAGE of extracts from wild-type (A), *ccs1*Δ (B), and *sml1*Δ *rad53*Δ (C) cells containing a Mac1-Myc fusion grown either in SD medium (lanes 2), in SD medium with 0.04, 0.1, or 0.3% MMS (A [lanes 3 to 5]), or in SD medium with 0.04 or 0.3% MMS (B and C [lanes 3 and 4]) for 1 h and then treated with AMS, followed by Western blot analysis to identify the Myc epitope. An extract from a mid-log-phase culture that was not treated with AMS was included as a control (lanes 1). Red, reduced; Ox, oxidized. (D) Steady-state Mac1 levels are not regulated in response to MMS. Extracts from cells expressing the Mac1-Myc fusion that had been grown in a range of MMS concentrations (0.04, 0.1, and 0.3%) for 1 h were analyzed by Western blot analysis to quantify the Myc epitope. Ponceau S staining of the polyvinylidene difluoride membrane was used as a loading control. (E) Extracts from cells expressing Mac1-Myc grown in 0.3% MMS (1 h) or 5 mM hydrogen peroxide (10 min) were incubated with or without DTT prior to the addition of AMS, followed by Western blot analysis to identify the Myc epitope. An extract from a mid-log-phase culture that was not treated with AMS was included as a control (lane 1). (F) Extracts from cells expressing the Mac1-Myc fusion grown in the presence or absence of 0.3% MMS were divided, and one aliquot was first treated with phosphatase (PPase) and then analyzed by reducing SDS-PAGE, followed by Western blot analysis to identify the Myc epitope. Modified Mac1 (Mod) was identified as a higher-molecular-weight form.

To analyze the oxidation state of Mac1, we used the alkylating agent 4-acetamido-4-maleimidylstilbene-2,2-disulfonic acid (AMS), which binds to reduced cysteine residues in proteins, causing increases in their molecular weights (35). Changes in the ability of Mac1 to bind AMS in extracts from cells grown with a range of MMS levels were monitored, and distinct forms of Mac1 were identified (Fig. 8A). In extracts from cells grown in SD medium, a condition under which Mac1 is inactive, the binding of AMS to Mac1 generated an increase in the molecular weight of Mac1 (Fig. 8A, 1st and 2nd lanes). Therefore, when grown in SD medium, Mac1 contains cysteine residues that are reduced and sufficiently solvent exposed to be accessible to alkylation. Under these conditions, however, Mac1 did not form a discrete band, consistent with the presence of multiple reduced forms of the protein that differed in the extent to which they were alkylated. In extracts from cells treated with a low level of MMS (0.04%), a condition that induces *CTR1* expression, Mac1 was identified as a discrete reduced band (Fig. 8A, 3rd lane). In response to a higher level of MMS (0.1%), which also induces *CTR1* expression, a discrete reduced form of Mac1 with an apparently reduced molecular weight was identified (Fig. 8A, 4th lane). Therefore, in response to low levels of MMS, Mac1 is modified so that some of its cysteine residues are no longer able to bind AMS. Treatment of cells with a level of MMS that inactivates Mac1 (0.3%) resulted in oxidized forms of the protein (Fig. 8A, 5th lane). Therefore, Mac1 undergoes changes in its redox state in response to MMS that correlate with its activity as a transcription factor.

The relationship between the activity of Mac1 and its oxidation state suggested that the loss of Mac1 activity in *ccs1*Δ or *sml1*Δ *rad53*Δ cells might be due to oxidization of Mac1, rendering it inactive in these strains. To test this hypothesis, the redox state of Mac1 in cells lacking Ccs1 or Rad53 and treated with MMS was analyzed. In each case, the redox profile of Mac1 in the *ccs1*Δ or *sml1*Δ *rad53*Δ mutants was similar to that identified in wild-type cells (Fig. 8B and C). Therefore, the loss of Mac1 function in cells lacking Ccs1 or Rad53 cannot be attributed to an oxidized inactive form of Mac1. The different forms of Mac1 identified using AMS do not represent degradation products, since Western blot analysis of wild-type extracts established that steady-state levels of Mac1 do not change in response to MMS (Fig. 8D).

To further analyze the redox nature of Mac1, the extent to which the oxidized inactive form of the protein can be reversed to a reduced form was analyzed. Cells were treated with either MMS (0.3%) or hydrogen peroxide (5 mM) to induce the oxidized forms of Mac1 (Fig. 8E). Cell extracts were then divided and incubated in the presence or absence of the reducing agent dithiothreitol (DTT) prior to incubation with AMS. Both MMS- and hydrogen peroxide-induced oxidized forms of Mac1 were reduced by treatment with DDT. However, in agreement with our previous experiments, MMS treatment resulted in a modification of Mac1 that resulted in less AMS binding (Fig. 8E, compare 3rd and 5th lanes). This modified form of Mac1 could also be identified by Western blot analysis of low-voltage-run reducing SDS-PAGE gels (Fig. 8F). Treatment of the cell extracts with phosphatase did not reverse this modification but resulted in the dephosphorylation of Rad53 in control experiments (Fig. 8F; also data not shown). Therefore, Mac1 undergoes reversible oxidation in response to high MMS levels that correlates with an inactive form of the protein. Conversely, treatment with low levels of MMS results in the reduction of Mac1 to an active form and a modification that does not involve phosphorylation.

### The mechanisms that regulate copper import in response to low copper levels or DNA damage are similar but not identical.

**Fig 9:**
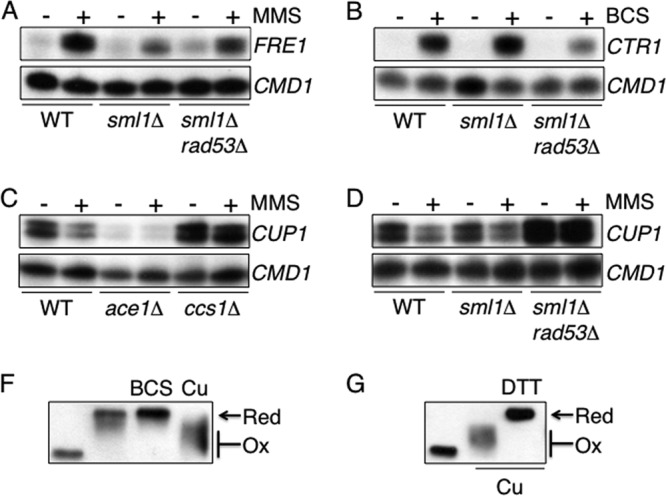
(A) The Mac1 target *FRE1* is regulated in response to MMS; (B) the response of *CTR1* to copper requires Rad53; (C and D) the regulation of *CUP1* requires Ccs1 (C) and Rad53 (D). *FRE1* and *CUP1* transcripts in cells treated with MMS (0.04%) (A, C, and D) and *CTR1* transcripts in cells grown with the copper chelator BCS (100 μM) and then treated with 0.04% MMS for 1 h (B) were analyzed by S1 nuclease protection assays using RNA isolated from wild-type, *sml1*Δ, and *sml1*Δ *rad53*Δ cells (A, B, and D) or wild-type, *ace1*Δ, and *ccs1*Δ cells (C). Calmodulin was used as a loading control (*CMD1*). (F) Mac1 undergoes changes in its redox state in response to copper. The oxidation state of Mac1 was analyzed by nonreducing SDS-PAGE of extracts from cells containing the Mac1-Myc fusion grown either in SD medium alone (2nd lane), in SD with the copper chelator BCS (3rd lane), or in SD with added copper (100 μM) (4th lane) and treated with AMS. Then Western blot analysis was performed to identify the Myc epitope. An extract from a mid-log-phase culture that was not treated with AMS was included as a control (1st lane). (G) Extracts from cells expressing Mac1-Myc grown in copper (100 μM) for 1 h were incubated with or without DTT prior to the addition of AMS, followed by Western blot analysis to identify the Myc epitope. An extract from a mid-log-phase culture that was not treated with AMS was included as a control (1st lane).

The finding that the response of *CTR1* to MMS requires both Mac1 and Sod1 activity suggested that there may be overlap between the low-copper- and DNA damage-sensing pathways in yeast. To analyze the relationship of these two signaling pathways further, we undertook the following experiments. To test if another Mac1 target, *FRE1*, is also regulated in response to MMS in a Rad53-dependent manner, *FRE1* transcript levels were analyzed in wild-type, *sml1*Δ, and *sml1*Δ *rad53*Δ cells treated with MMS. In wild-type cells, *FRE1* transcripts were induced in response to MMS, similarly to *CTR1* transcripts (Fig. 9A). However, in contrast to the *CTR1* expression profile, full induction of *FRE1* was dependent on Sml1; *FRE1* transcripts were induced to low levels in both the *sml1*Δ and *sml1*Δ *rad53*Δ strains (Fig. 9A). This is consistent with a link between the activity of the iron-containing ribonucleotide reductase and the regulation of metal reductase activity on the cell surface. To test whether Rad53 is involved in low-copper sensing, *CTR1* transcripts were analyzed in wild-type, *sml1*Δ, and *sml1*Δ *rad53*Δ cells that had been grown in the presence or absence of the copper chelator BCS (Fig. 9B). In wild-type and *sml1*Δ cells, *CTR1* transcript abundance increased, as expected, in response to copper limitation; however, levels were lower in the *sml1*Δ *rad53*Δ strain (Fig. 9B). Therefore, Rad53 is required for the full induction of *CTR1* in response to MMS and, separately, copper limitation.

In S. cerevisiae, the activities of Mac1 and the high-copper sensor AceI are coordinated, so that their target genes are oppositely regulated in response to copper levels (22, 23). To determine if Mac1 and AceI targets are regulated similarly in response to MMS, *CUP1* transcript levels were quantified in cells treated with MMS (Fig. 9C). In wild-type cells, MMS treatment resulted in a reduction in the level of *CUP1* transcripts; however, *CUP1* transcript levels were higher in both MMS-treated and untreated *ccs1*Δ cells (Fig. 9C). Therefore, as with copper-responsive gene regulation, *CTR1* and *CUP1* transcripts are oppositely regulated in response to MMS. In addition, the wild-type regulation of *CUP1* requires the activity of the copper chaperone Ccs1. A possible role for Rad53 in the regulation of the *CUP1* metallothionein gene was also tested. In cells lacking Rad53, *CUP1* transcript levels were higher than in wild-type cells and did not change in response to MMS treatment (Fig. 9D). Therefore, as with *CTR1*, the regulation of *CUP1* in response to MMS requires the activities of Ccs1 and Rad53.

The changes identified in the oxidation state of Mac1 in response to MMS suggested that similar changes might take place in response to copper and might therefore reflect the mechanism by which copper is sensed by yeast. The extent to which Mac1 undergoes changes in its oxidation state in response to copper levels was therefore analyzed using the AMS protocol. In extracts from cells grown in the copper chelator BCS, a condition that activates *CTR1* expression, Mac1 was found in a discrete reduced form (Fig. 9F). Conversely, in extracts from cells treated with a high level of copper that represses *CTR1* expression, Mac1 was found in a range of oxidized forms (Fig. 9F). These forms do not represent Mac1 degradation products, since the oxidized form could be reduced with DTT prior to AMS binding (Fig. 9G). Therefore, the redox profile of Mac1 in response to copper is similar to that seen in response to MMS: a reduced form correlates with the active form of the protein.

## DISCUSSION

Through a genome-wide genetic screen, we have identified a novel regulatory relationship between copper metabolism and the DNA damage-sensing pathway in yeast. Mutants lacking genes involved in DNA repair were found to express high levels of metal reductase activity on the cell surface. In response to DNA-damaging agents, copper import genes are induced and a copper-sequestering metallothionein gene is repressed, suggesting a need for copper under these conditions. This is consistent with the sensitivity of mutants lacking the copper importer Ctr1 or the low-copper-sensing transcription factor Mac1 to growth on MMS. Regulation of the copper genes is dependent on the copper-responsive transcription factors, the copper chaperone Ccs1, and the checkpoint kinase Rad53 but is independent of the Mec1 DNA damage-signaling proteins Mec1, Dun1, and Crt1. The reason why genes involved in copper homeostasis are regulated in this manner is suggested by the aberrant response of a Rad53 target to MMS in copper-starved cells. Under these conditions, the induction of the *RNR3* gene is repressed unless cells express an iron-containing superoxide dismutase, consistent with the notion that reduced copper levels result in a decrease in Sod1 activity that adversely impacts on Rad53 signaling.

A regulatory link between copper homeostasis and the DNA damage pathway in yeast is supported by analysis of the oxidation state of Mac1. Mac1 undergoes changes in its redox state in response to MMS or copper levels that correlate with its activity. A simple model is that critical cysteine residues within Mac1 are regulated via reversible oxidation. To test this model, we have attempted to analyze the oxidation state of Mac1 variants with substitutions of the copper-sensing residues in the C1 sensing domain. Although these mutations generate constitutively active sensors, as expected, the steady-state levels of these Mac1 variants are considerably lower than wild-type levels, which has precluded analysis of Mac1 using the AMS protocol (data not shown). We accept that future evidence of cysteine residue involvement in the gel mobility changes in Mac1 that we have observed is required to support the redox model of Mac1 regulation. Assuming that our model is correct, it is noteworthy that the endogenous levels of reactive oxygen species in yeast are increased in mutants lacking DNA damage genes, which could account for the results of our genetic screen (58). It remains to be seen how low copper levels or low MMS levels cause the reduction of Mac1. One possibility is that a complex relationship between copper, DNA damage, and reactive oxygen species levels results in subtle changes in fluxes through the glycolytic and pentose-phosphate pathways (62, 63). The glycolytic enzyme glyceraldehyde-3-phosphate dehydrogenase (GAPDH) is inhibited by oxidative stress, resulting in increased flux through the pentose-phosphate pathway and the production of reducing equivalents via NADPH (62, 63). Redox sensors may respond to these changes and interact with Mac1 to control its activity by a mechanism analogous to the mechanism that regulates the redox-responsive transcription factor Yap1.

Although we have identified a novel aspect of Mac1 regulation, some aspects of this mechanism are not clear. Under conditions where Mac1 is transcriptionally inactive, it was found to exist in a range of different reduced forms. MMS treatment also resulted in the modification of some cysteine residues in Mac1, preventing their subsequent reduction by DTT or alkylation by AMS. This could result from overoxidation of cysteine residues to sulfinic or sulfonic acid. Alternatively, treatment of cells with MMS may result in the methylation of some cysteine residues in Mac1. Adaptive responses to methylating agents such as MMS are found in many bacteria, suggesting that microorganisms regularly encounter these compounds (64). Precedence for such a mechanism is found in studies of the transcription factor Ada from E. coli, which regulates DNA damage genes and is activated by the methylation of a critical cysteine residue (65). Again, analysis of different Mac1 cysteine mutants will be needed in order to determine which residues are important for generating the different Mac1 forms identified in this study.

The similar changes in the redox state of Mac1 in response to copper and MMS suggest that there is mechanistic overlap between these two signaling pathways. Copper import (*CTR1*) and copper sequestration (*CUP1*) are regulated oppositely in response to both MMS and copper by a process that requires Sod1 and Rad53 activity. In addition, bacterial SodB, which lacks a nuclear localization signal, is unable to restore the response of Mac1 to MMS in a mutant lacking the Ccs1 chaperone (data not shown), in agreement with the previous finding that nuclear Sod1 is required for copper sensing (31). In contrast, however, the Mac1^up^ allele, which does not respond to copper, is repressed by high levels of MMS, a finding consistent with the existence of some mechanistic differences between the two signaling pathways. What is clear is that the signaling pathway that regulates the *RNR* genes in response to MMS is distinct from that which regulates copper homeostasis. While the entire Mec1 pathway is required for *RNR* regulation, only Rad53 is involved in copper regulation. Furthermore, regulation of *RNR3* can be restored by cytosolic SodB, consistent with the presence of a cytosolic defect in DNA damage sensing in the *ccs1*Δ mutant rather than the established nuclear defect in copper sensing.

**Fig 10:**
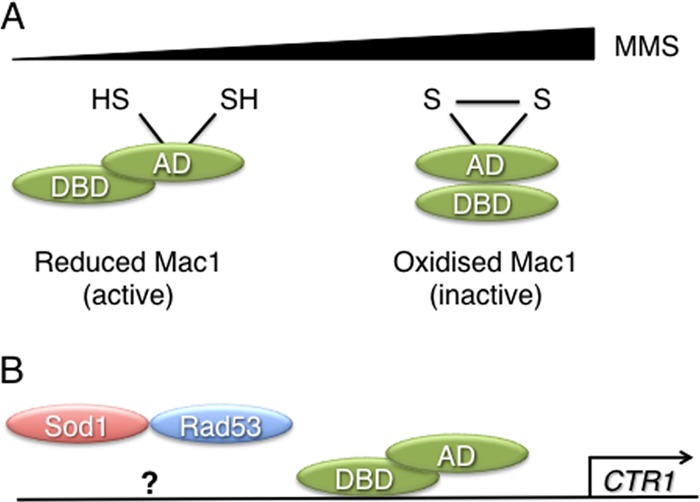
Schematic representation of MMS sensing by Mac1. (A) In response to low MMS levels, cysteine residues in Mac1 are predominantly reduced, although some are modified, perhaps by methylation. High levels of MMS result in the reversible oxidation of cysteine residues in the activation domain (AD) of Mac1, resulting in repression of the activity of Mac1 as a transcription factor. Only 2 of the 10 cysteine residues in the C1 and C2 domains are shown. (B) The reduced form of Mac1 correlates with the active form of the transcription factor. Both Sod1 and Rad35 are required for an unknown aspect of the mechanism controlling *CTR1* expression.

The control of *CTR1* expression appears to involve two distinct mechanisms. Sod1 and Rad53 activity are both necessary for the control of copper gene expression; however, they do not regulate redox changes in Mac1 that correlate with its activity, since these occur in mutants lacking either Sod1 or Rad53. At present it is not clear why Mac1 is inactive in these cells. Mac1 also is not regulated in response to changes in Sod1 activity, since Sod1 activity remains constant in response to levels of MMS to which Mac1 responds and in a mutant lacking Rad53. This is consistent with the notion that Mac1 signaling is independent of changes in internal copper levels and with our finding that cellular copper levels do not change in response to MMS (data not shown). We accept, however, that the measurement of cellular copper levels and Sod1 activity in whole-cell extracts will likely not detect changes in nuclear levels, which may be mechanistically important. Based on our findings, we present a model in which the activity of Mac1 is dependent separately on its oxidation state and the checkpoint kinase Rad53 (Fig. 10). In response to low levels of MMS, regulatory cysteine residues in Mac1 are predominantly reduced, although some residues are modified. High levels of MMS result in the oxidation and inactivation of Mac1, consistent with a lack of *CTR1* repression under anaerobic growth conditions (Fig. 10A). The reduced and partially modified form of Mac1 is required for the transcription of Mac1 target genes together with Rad53 and Sod1 (Fig. 10B). In cells lacking Sod1 activity, Rad53 is inactive, preventing the response of Mac1 to MMS or changes in copper levels. Our preliminary studies have also identified changes in the oxidation state of the transcription factor AceI in response to MMS, suggesting that it may also be controlled by a similar mechanism.

Earlier studies of low-copper sensing in yeast modeled Mac1 function based on the binding of copper to this transcription factor. The finding that Mac1 responds to conditions other than changes in copper levels and exists in different redox forms that relate to its activity suggests more-complex mechanisms of control. Specifically, Mac1 may respond to cellular redox changes via the reversible oxidation of critical cysteine residues. If the mechanism of copper sensing in yeast is conserved in higher eukaryotes, the inappropriate import of copper when cells experience DNA damage or oxidative stress may contribute to disease. A full understanding of copper sensing in yeast should reveal novel interactions between copper, oxidative stress, and genome maintenance, with possible implications for human health.

## Supplementary Material

Supplemental material
