# Supplementary material for: The Yeast Copper Response Is Regulated by DNA Damage
Source: Mol Cell Biol. 2013 Oct;33(20):4041–50. doi: 10.1128/MCB.00116-13 (PMC3811678; doi:10.1128/MCB.00116-13)
Supplement: Supplemental material [file supp_33_20_4041__index.html]

Supplemental material 

# The Yeast Copper Response Is Regulated by DNA Damage

## Supplemental material

**Files in this Data Supplement:**

- Supplemental file 1 -

  Table S1 (Yeast genes whose deletion resulted in high cell surface metal reductase activity)

  XLSX, 50K
